# Back to the USSR: How Colors Might Shape the Political Perception of East versus West

**DOI:** 10.1177/2041669516676823

**Published:** 2016-11-16

**Authors:** Fabian Gebauer, Marius H. Raab, Claus-Christian Carbon

**Affiliations:** Department of General Psychology and Methodology, University of Bamberg, Bamberg, Germany; Research group EPÆG (Ergonomics, Psychological Æsthetics, Gestalt), Bamberg, Germany; Bamberg Graduate School of Affective and Cognitive Sciences (BaGrACS), Bamberg, Germany

**Keywords:** political perception, colors, Ukraine Crisis, stereotypes, information processing

## Abstract

People typically process information to confirm their prior held attitudes and stereotypes. As the political relations between NATO and Russia have distinctively drifted apart in recent years, we were interested in how far old-established color depictions referring to the Cold War’s demarcations (USSR = red; NATO = blue) might reinforce people’s political perception of an East versus West antagonism nowadays. Participants received a fabricated news article in which both world powers were either depicted on a map as Russia = red and NATO = blue or vice versa (Study 1). Testing a different sample in Study 2, we fully removed color assignments and used hachured distinctions or no distinctions at all. We revealed that perceived political distance between both sides increased particularly for participants with negative attitudes toward Russia, but only when Russia was depicted in red. Thus, colors referring to the old-established Cold War patterns can indeed shape the political perception and reinforce stereotypical East versus West thinking.

From Stettin in the Baltic to Trieste in the Adriatic, an iron curtain has descended across the Continent.

—Winston Churchill (1946)

Although political systems and territorial boundaries have shifted since the seminal speech by Winston Churchill, his words seem as topical as ever since the fall of the Iron Curtain 26 years ago. Along with the annexation of Crimea by the Russian Federation, threatening *East versus West* depictions returned in common news coverage, often accompanied by illustrations visualizing old-established Russia versus NATO stereotypes (referring to the former USSR with red and to the NATO member states with blue; Gebauer, Raab, & Carbon, 2016). In fact, research has shown that color can increase the perceived polarization between Democrats and Republicans among the American electorate (Rutchick, Smyth, & Konrath, 2009), and that subtle visual metaphors depicted on a map can affect people’s attitudes (Schoormans, Carbon, & Gattol, 2011).

In many real-world contexts, like when reading a newspaper or watching the newscast, people do not process information in a neutral way; instead, they search for information that confirms their prior held attitudes and stereotypes (Kruglanski, 2004), even affecting a mental distance gap between East and West (Carbon & Leder, 2005). Following these ideas, we aimed at investigating how far participants’ perception—especially for those holding strong attitudes against Russia—of the political relationship between Russia and the NATO members might be affected by the mere visualization of the territories of both power blocks. In Study 1 (*N* = 75; 39 male; *M*_age_ = 23.1 years, *SD* = 2.4), we implemented a map either visualizing the typical colorization used during the Cold War, that is, Russian territory in red and NATO territory in blue, or showing an inversed and thus atypical color assignment (Figure 1, left column). In Study 2 (*N* = 70; 43 female; *M*_age_ = 23.7 years, *SD* = 2.9), we removed the color classifications and replaced them either with hachured distinctions or no graphical distinction at all (Figure 1, right column). All participants were invited to read and to evaluate a fabricated news article that was the same in both studies and conditions and only differed in the type of map embedded. The text described the armament of Russia as well as of the NATO states in a matter-of-fact style, with an equal amount of text for both sides. A pretest (*N* = 20; 14 female; *M*_age_ = 28.2 years, *SD* = 9.0) showed no significant difference between the level of induced threat regarding the written description of Russia (*M* = 3.30, *SD* = 1.56) and the NATO states (*M* = 3.75, *SD* = 1.55), *F*(1,19) = 1.03, *p* = .324, $\eta_{\text{p}}^{2}$ = .05. Before reading the fabricated news article, all participants in both studies, which were conducted simultaneously in July 2015 to ensure a maximum of comparability, were asked to rate on a 6-point semantic differential “Who is to blame for the Ukraine Crisis?” (NATO vs. Russia). Blame scores did not differ significantly in any of the conditions used in Study 1 and Study 2, *F*(3,141) < 1, *p* = .731, $\eta_{\text{p}}^{2}$ = .009. After participants read the article, they were asked to rate the perceived political distance between both power blocks using the following item: “The political distance between the NATO states and Russia seems extremely strong to me” (1 = *strongly disagree*, 7 = *strongly agree*). As a last step, all participants were debriefed in Study 1 as well as in Study 2.

**Figure 1.**
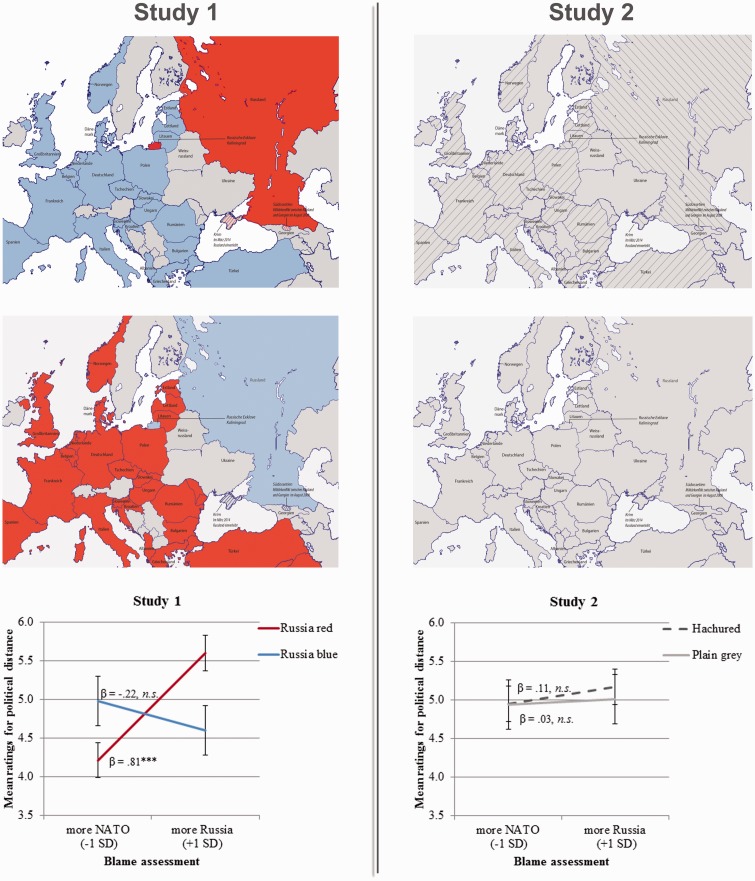
The maps used for Study 1 (left column) and Study 2 (right column). Bottom row showing mean ratings for the perceived political distance between the NATO and Russia by map condition (Study 1; Russia = red vs. Russia = blue and Study 2; hachured vs. plain grey) and prior held blame assessments (continuous and centered variable) for the Ukraine Crisis. Error bars indicate ± 1 standard error of the mean (*** indicates *p* < .001).

In Study 1, regressing political distance scores onto *map condition* (Russia red vs. Russia blue; dummy coded), *blame assessments* (continuous and centered), and their interaction showed no main effect for *map condition*, β = .12, *SE* = .32, *t*(71) < 1, *p = .*708, *R*^2 ^= .002, and none for *blame assessment*, β = .29, *SE* = .20, *t*(71) = 1.43, *p* = .157, *R*^2 ^= .069. The two-way interaction, however, turned out to be significant, β = 1.03, *SE* = .34, *t*(71) = 2.59, *p* = .012, *R*^2 ^= .080 (Figure 1; bottom of left column). The interactions in Figure 1 are plotted at one standard deviation above and below the centered mean of the blame assessment scores (Aiken & West, 1991). Simple slope analysis revealed that blame assessments (Russia high) significantly and positively predicted political distance scores in the Russia = red condition, β = .81, *SE* = .23, *t*(71) = 3.46, *p* < .001, but not in the Russia = blue condition, β = −.22, *SE* = .32, *t*(71) < 1, *p* = .492. Additionally, among participants with high blame assessments toward Russia, political distance scores were higher in the Russia = red condition, β = 1.01, *SE* = .77, *t*(71) = 2.14, *p* = .036. There was no simple effect of condition, in contrast, among participants with low blame assessments toward Russia, β = −.76, *SE* = .47, *t*(71) = 1.63, *p* = .108.

Study 2 showed that regressing political distance scores onto *map condition* (hachured vs. plain gray; dummy coded), *blame assessments* (continuous and centered), and their interaction revealed neither a main effect for *map condition*, β = .09, *SE* = .32, *t*(66) < 1, *p = .*792, *R*^2 ^= .001, nor for *blame assessments*, β = .07, *SE* = .17, *t*(66) < 1, *p = .*661, *R*^2 ^= .003, and additionally no interaction effect, β = .08, *SE* = .33, *t*(66) < 1, *p = .*821, *R*^2 ^< .001. Consequently, simple slope analyses showed no significant effects, all *t*_s_ < 1 (Figure 1; bottom of right column).

Results indicate that colors depicting the old-established Cold War patterns can indeed affect participants’ political perception of an East versus West conflict that has come full circle. Such color schemes can induce a higher perceived political distance, especially for those who strongly blame Russia for this crisis. Thus, these depictions might not affect people per se but can confirm and reinforce prior held attitudes—whether the strong effect of the color red in our study can be attributed to a kind of red-negative-associations or stereotypical red-Soviet Union-associations cannot fully be decided within the scope of the current study; also note that although red = negative stereotypes have been documented so far, other authors also claim positive relations with the color and others fail to find any main effect of red at all (Hesslinger, Goldbach, & Carbon, 2015). To create a better understanding of colors and political thinking, future research might extend these findings to other antagonistic country pairs (e.g., India and Pakistan^1^), where neither is associated with the color red. However, our findings are a first step toward a fairly undiscovered field of psychological research and are in accordance with former findings showing that prior attitudes can bias information processing (Kruglanski, 2004) and mental representations (Carbon & Leder, 2005; Hesslinger et al., 2015). Additionally, findings might help to better understand the power of specific depictions in news coverage by applying these ideas to the domain of color-induced stereotypes.

## Note

1 We would like to thank one of the anonymous reviewers for pointing out this excellent idea.
